# Sequential and combinatorial roles of *maf* family genes define proper lens development

**Published:** 2007-01-16

**Authors:** Hasan Mahmud Reza, Atsuyo Urano, Naoko Shimada, Kunio Yasuda

**Affiliations:** Graduate School of Biological Sciences, Nara Institute of Science and Technology, Takayama, Ikoma, Japan

## Abstract

**Purpose:**

Maf proteins have been shown to play pivotal roles in lens development in vertebrates. The developing chick lens expresses at least three large Maf proteins. However, the transcriptional relationship among the three large *maf* genes and their various roles in transactivating the downstream genes largely remain to be elucidated.

**Methods:**

Chick embryos were electroporated with wild-type *L-maf*, *c-maf,* and *mafB* by in ovo electroporation, and their effects on gene expression were determined by in situ hybridization using specific probes or by immunostaining. Endogenous gene expression was determined using nonelectroporated samples.

**Results:**

A regulation mechanism exists among the members of *maf* family gene. An early-expressed member of this gene family typically stimulates the expression of later-expressed members. We also examined the regulation of various lens-expressing genes with a focus on the interaction between different Maf proteins. We found that the transcriptional ability of Maf proteins varies, even when the target is the same, in parallel with their discrete functions. L-Maf and c-Maf have no effect on E-cadherin expression, whereas MafB enhances its expression and thereby impedes lens vesicle formation. This study also revealed that Maf proteins can regulate the expression of gap junction genes, *connexins*, and their interacting partner, major intrinsic protein (MIP), during lens development. Misexpression of L-Maf and c-Maf induces ectopic expression of Cx43 and MIP; in contrast, MafB appears to have no effect on Cx43, but induces MIP significantly as evidenced from our gain-of-function experiments.

**Conclusions:**

Our results indicate that large Maf function is indispensable for chick lens initiation and development. In addition, L-Maf positively regulates most of the essential genes in this program and directs a series of molecular events leading to proper formation of the lens.

## Introduction

The vertebrate lens develops from the overlying surface ectoderm and becomes polarized into anterior cuboidal epithelium and posterior fiber cells. Cell proliferation and differentiation initiate at the equatorial region and persist throughout life. The posterior cells exhibit an array of specific gene expression constituting the lens structural proteins by the coordinated action of growth and transcription factors. Transcription factors serve as the fundamental basis for making an organ by their regulatory functions, and this has been illustrated for lens development in many species. The key role played by transcription factors during morphogenesis is to direct gene expression and cell differentiation. As development progresses, members of a particular family or different regulator proteins are expressed with overlapping as well as divergent patterns. The integrated functions of these molecules result in the high level expression of crystallins and noncrystallin genes. The accumulation of crystallins, which confer transparency and refractivity on the lens, is remarkably observed in differentiated fiber cells [1]. Studies from a wide range of organisms have demonstrated that several transcription factors, Pax6, Sox1, 2, 3, Prox1, Six3 and Maf, are important for lens formation [2-6].

Three large Maf family proteins, L-Maf, c-Maf, and MafB, have been found to take part actively in the lens development program (for a review, see [7]). An endogenous order of expression for these three genes demonstrates that L-Maf is expressed first, followed by c-Maf and then MafB [7]. Maf response elements (MAREs) within the promoter/enhancer region of the *crystallin* gene family have been identified, and both in vitro and in vivo studies have shown that crystallins are regulated by Maf through these regulatory sites [4,8,9]. Since the expression of crystallins is temporally and spatially regulated, and the cis-acting sites are arranged differently in their regulatory sequences, different interactions are possible [10]. As most crystallins have the MARE sequence, Maf proteins are considered to be an important class of regulators that control the transcriptional activation of these genes based on the availability of each Maf member. Therefore, different Maf members, in association with ubiquitously expressed factors, possibly result in the diversity of crystallin expression [7,11]. Studies on knockout mice for *c-maf* have revealed a hollow lens with reduced crystallin expression, suggesting an important role for Maf in lens development [12-14]. However, these studies have shown that lens initiation in mice is not dependent on Maf activity; therefore, the primary role of Maf is thought to be in later lens fiber differentiation. On the other hand, the failure of lens induction caused by dominant-negative L-Maf expression in the presumptive lens ectoderm indicates that L-Maf plays a critical role in the chick lens induction program [6]. This finding suggests a discrepancy between Maf functions in different organisms. It has recently been demonstrated that MAF mutation (substitution of arginine with proline at residue 288) results in pulverulent cataract, microcornea, iris coloboma, and anterior segment dysgenesis in human [15]. This mutation has been shown to eliminate the transcriptional activity of Maf [16]. Closely linked with this, the mouse mutation R291Q also results in cataract [17]. Previously, we showed by transfection in cultured cells that L-Maf, c-Maf, and MafB are able to induce δ-crystallin expression to different degrees [8]. Although we successfully detected all three large Mafs in the developing chick lens, we still lack evidence about the regulatory relationships within this family and with many of their downstream targets. Several noncrystallin genes expressed in lens during development have been suggested to contribute to the formation of lens cytoskeletal structure. CP49 and CP95/115 are lens-specific beaded filaments that form a meshwork underneath the plasma membrane of the lens fiber cells [18-20]. A recent study with *c-maf*-knockout mice reported that the expression of CP49 and CP115 is not absolutely regulated by c-Maf but its indirect involvement has been suggested [21]. Expression patterns of different cadherins indicate that this class of adhesion molecule plays important roles in lens development [22]. Besides cell adhesion, for development, differentiation and growth of the lens, cell-to-cell communication is another crucial aspect, and is performed by gap junctions [23,24]. Overexpression of Cx45.6 in chick lens primary cultures stimulates lens cell differentiation coupled with an enhancement in the expression of δ-crystallin and major intrinsic protein (MIP) [25]. MIP belongs to the aquaporin family of water channels and it appears most abundantly as a membrane protein in lens fiber [26]. It has been shown that MIP interacts directly with the intracellular loop domain of lens fiber-specific Cx45.6, but has no effect on gap junction-mediated intercellular communication [24].

Elucidation of upstream factors responsible for specific expression of all these genes is important to understand morphogenesis of the lens. We performed a series of gain-of-function experiments to establish a regulatory link among the members of *maf* family genes during eye development by in ovo electroporation in chick embryos. We found that early-expressed Maf proteins exert a forward transcriptional control on later-expressed members. Notably, MafB, which is expressed much later, exhibits a negative effect on L-Maf and c-Maf expression. Large Maf proteins share some common activities in transactivating downstream genes, yet their ability to do so varies and is controlled by spatially and temporally specific mechanisms. We have also found distinct functions of Maf proteins in activating cadherin, MIP, and connexins in eye lineage. Regulation of gap junction and water channel genes by Maf is a novel finding that enables us to consider Maf as a regulator of a wide range of genes essential for vertebrate lens development. Our results demonstrate that *maf* genes are hierarchically regulated within the family, and that their redundant and discrete functions with respect to the expression of lens *crystallin* and noncrystallin genes determine lens induction and fiber differentiation programs. We conclude that the involvement of large Maf proteins is critical for chick lens development.

## Methods

### Plasmid construction

Chicken wild-type *L-maf*, *c-maf*, and *mafB*, and dominant-negative *L-maf* plasmids have been described previously [4,6,8].

### Embryo staging

Fertilized white Leghorn eggs were incubated at 38.5 °C, and the embryos were staged according to Hamburger and Hamilton [27].

### In ovo microelectroporation

In ovo microelectroporation was performed essentially as described [4]. Plasmid DNA of interest at a concentration of 5 μg/μl was electroporated into chick embryos at stage 9-10, together with the plasmid pCAGGS-GFP to monitor efficiency of incorporation of DNA into embryos.

### Tissue preparation

Embryos were fixed overnight in phosphate buffered saline (PBS) containing 4% paraformaldehyde (PFA) and 0.1 M MOPS, 2 mM EGTA, 1 mM MgSO4 (1% MEM), washed with PBS, treated successively with 10%, 20%, and 30% sucrose in PBS, dipped in OCT compound to remove excess sucrose, and finally embedded in fresh OCT medium. Embedded embryos were then sectioned at a thickness of 10 μm. Tissue sections were mounted on glass slides and air-dried for 2 h. Similarly, embryos after whole-mount immunostaining were again fixed in 4% PFA and 0.1% glutaraldehyde in PBS for 30 min at room temperature and dehydrated through a graded series of ethanol. Dehydrated embryos were treated with xylene twice, for 5 min each and incubated in paraffin for 2 h. Next, they were embedded in fresh paraffin and sectioned (10 μm) with a microtome.

### Whole-mount and cryosection immunostaining

Whole-mount immunostaining was performed as described in reference [28]. A δ-crystallin monoclonal antibody provided by G. Eguchi (President, Kumamoto University, Kumamoto 860-8556, Japan) [29] was used at 1:20 dilution in 2% BSA. Anti-E-cadherin and anti-Pax6 monoclonal antibodies were obtained from Transduction Laboratories (Lexington, KY) and DSHB (Iowa City, IA), respectively, and used at 1:500 dilution in 2% BSA. For whole-mount staining, δ-crystallin immune complexes were detected with antibodies to mouse IgG conjugated with horseradish peroxidase (DAKO, Hamburg, Germany) by diaminobenzidine. For cryosections, immunofluorescence cell staining was carried out according to the protocol described in Research Applications of Santa Cruz Biotechnology, 1994, with slight modification. L-Maf and Sox2 immune complexes were detected with antibody to rabbit IgG conjugated with AlexaFluor 594; Pax6, δ-crystallin and E-cadherin immune complexes were detected with AlexaFluor 594 conjugated antimouse IgG. E-cadherin immune complex was also detected with AlexaFluor 488 conjugated antimouse IgG (green). Anti-rat IgG-Cy3 was used to detect N-cadherin complexes.

### Whole-mount and cryosection in situ hybridization

Whole-mount and cryosection in situ hybridization were performed as described earlier [30]. Antisense RNA probes were prepared using digoxigenin-UTP (Boehringer Mannheim) by in vitro transcription of gene-specific fragments from cDNA templates with T3 or T7 RNA polymerase (Promega). Riboprobes for L-Maf, c-Maf, MafB [8] and Six3 [6] have been described previously. Plasmids used to generate probes for Cx43 [31] and MIP (full-length, GenBank AY078179) were kindly provided by Drs. V.M. Berthoud (Department of Pediatrics, University of Chicago, Chicago, IL) and J.X. Jiang (Department of Biochemistry, University of Texas Health Science Center, San Antonio, TX), respectively. A fragment of chick *Cx45.6* cloned in pBluescript was used to make an appropriate riboprobe. An alkaline phosphatase-conjugated sheep antidigoxigenin antibody was used for the detection of the labeled nucleic acids. For double in situ hybridization, fluorescein-labeled c-Maf probe and antifluorescein antibody were used.

## Results

### Large *maf* gene expression in developing lens

**Figure 1:**
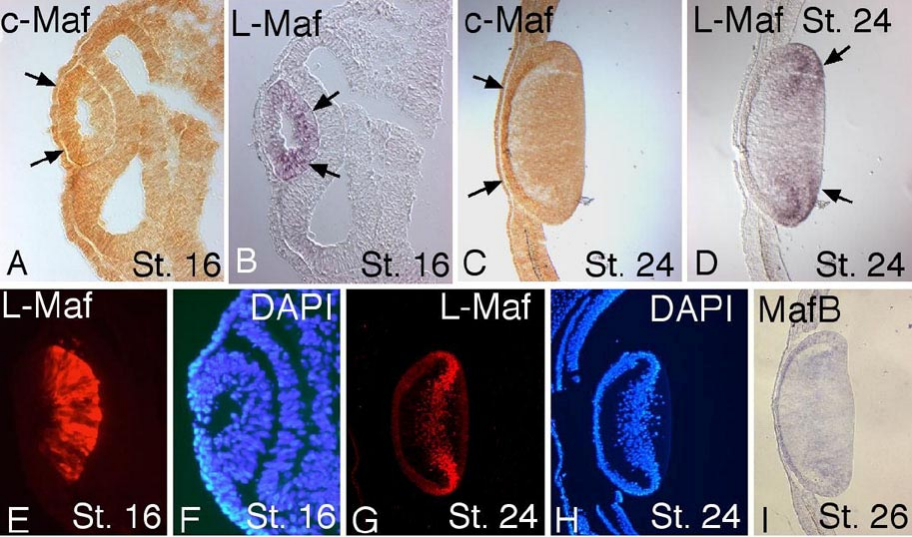
Endogenous expression patterns of three large Mafs. Wild-type embryos were isolated at stage 16 and 24, fixed, and cryosections were prepared. Through the use of double in situ hybridization, c-Maf and L-Maf mRNAs were detected on the same samples (**A**-**D**). In both stages, c-Maf was preferentially localized in the developing lens epithelum (**A** and **C**, arrows), whereas L-Maf was strongly localized in the fiber cells (**B** and **D**, arrows). Immunostaining using anti-L-Maf antibody mimicked the same result (**E**-**H**) as that observed by in situ hybridization for L-Maf. A low expression of MafB was visualized in all lens cells at stage 26 (**I**). DAPI shows cell nuclei (**F**,**H**). This figure is representative of at least three independent experiments.

Both L-Maf and c-Maf are expressed in the developing chick lens earlier than MafB [7]. To examine the spatial expression of L-Maf and c-Maf in more detail, we performed a double in situ hybridization, using probes for L-Maf and c-Maf, on tissue sections collected from stage 16 and 24 embryos. We observed that c-Maf is relatively localized to the prospective epithelial cells (Figure 1A,C), whereas L-Maf is abundant in fiber cells (Figure 1B,D), although a low level of expression for both c-Maf and L-Maf can be discerned in all lens cells. Immunostaining using L-Maf antibody also yielded similar results (Figure 1E,G). We also examined the expression of MafB by in situ analysis. MafB expression was not observed at stage 16, but a low level was detected in all lens cells at stage 26 (Figure 1I).

### Transcriptional regulation among the Maf members

**Figure 2:**
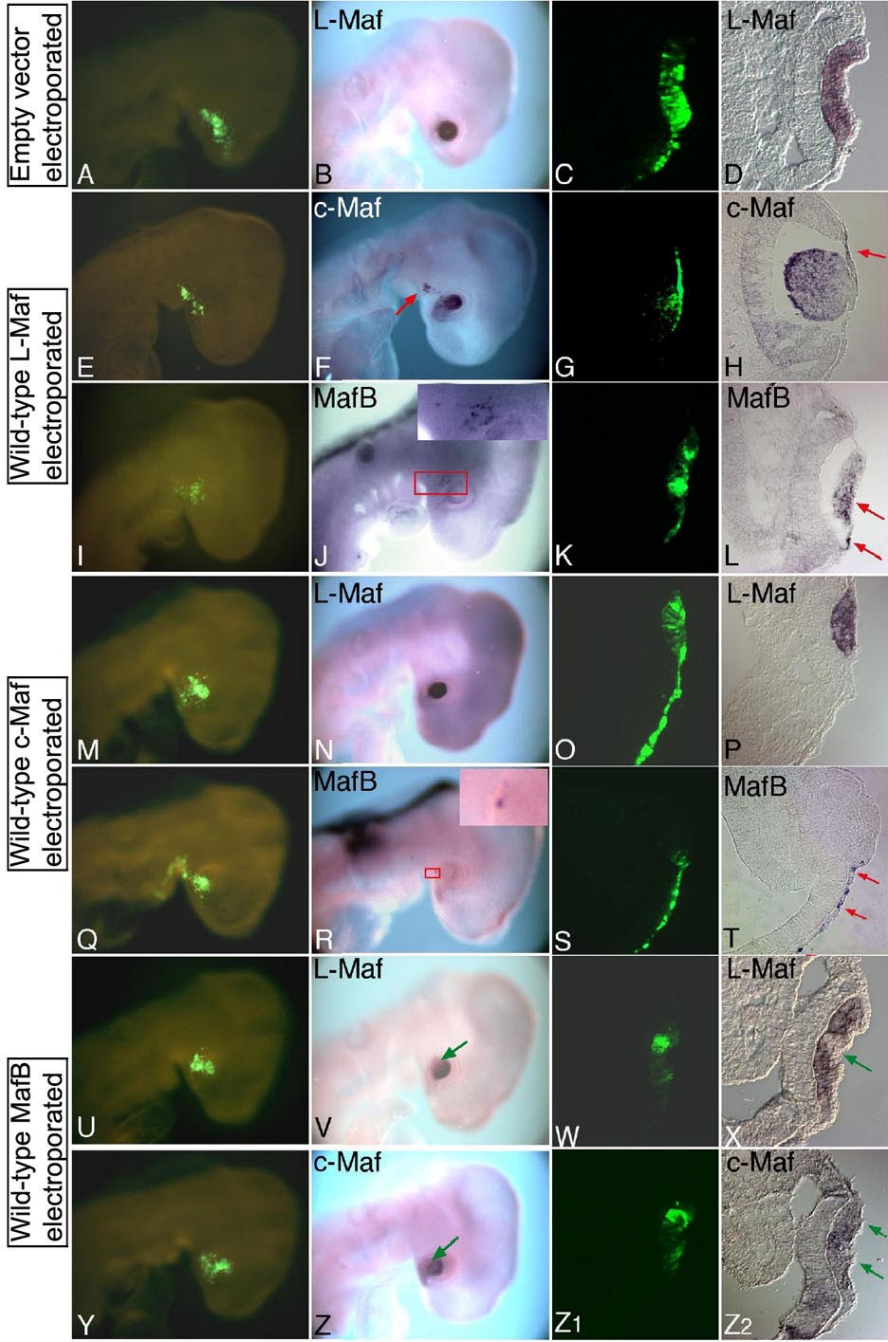
*maf* family gene members are mutually regulated during lens development. Chick embryos were electroporated with wild-type empty vector (**A**-**D**), L-Maf (**E**-**L**), c-Maf (**M**-**T**), and Maf B (**U**-**Z2**) in the overlying ectoderm at stage 10 and examined at stage 16 by in situ hybridization. Whole-mount and cryosection in situ hybridizations revealed ectopic c-Maf (**F** and **H**, red arrows) and MafB (**J**, red box shown in the inset; **L**, red arrows) expression by L-Maf overexpression. Note that endogenous MafB is not expressed in lens cells at this stage. Similarly, c-Maf induced MafB (**R**, red box shown in the inset; **T**, red arrows) but not L-Maf. On the other hand, MafB downregulated the expression of both L-Maf (**V** and **X**, green arrows) and c-Maf (**Z** and **Z2**, green arrows). A control experiment with empty vector showed no effect on normal expression of L-Maf (**B** and **D**). GFP fluorescence shows the electroporated area (1st and 3rd columns). This figure is representative of at least three independent experiments. For whole mounts, five to six embryos were used each time.

To address transcriptional relationships in lens lineage among the members of Maf family transcription factors, we electroporated chick embryos with L-Maf, c-Maf, and MafB in independent experiments (Figure 2). Subsequent analyses by whole-mount and cryosection in situ hybridizations yielded results that differed depending upon the gene electroporated. Control experiments, in which the empty vector was electroporated into the embryos, did not show any change in the expression of L-Maf (Figure 2A-D) or c-Maf and MafB (data not shown). When we analyzed L-Maf-electroporated embryos for the expression of c-Maf and MafB (Figure 2E-L), we observed high levels of mRNAs in the population expressing wild-type L-Maf (Figure 2F,H and Figure 2J,L, respectively; arrows and box in the inset), suggesting that L-Maf functions upstream of other *maf* genes. Similarly, misexpression of c-Maf (Figure 2M-T) induced MafB (Figure 2R,T, arrows and box in the inset); however, neither ectopic expression nor downregulation of L-Maf by c-Maf was observed (Figure 2N,P). In contrast, when MafB was misexpressed (Figure 2U-Z2), no ectopic expression of L-Maf or c-Maf was visualized. In addition, MafB suppressed the transcription of L-Maf and c-Maf in lens cells (Figure 2V,X and Figure 2Z,Z2, respectively; green arrows). Contralateral nonelectroporated eyes of all the embryos showed normal expression of the genes analyzed (data not shown).

### L-Maf is indispensable for lens induction in chick

**Figure 3:**
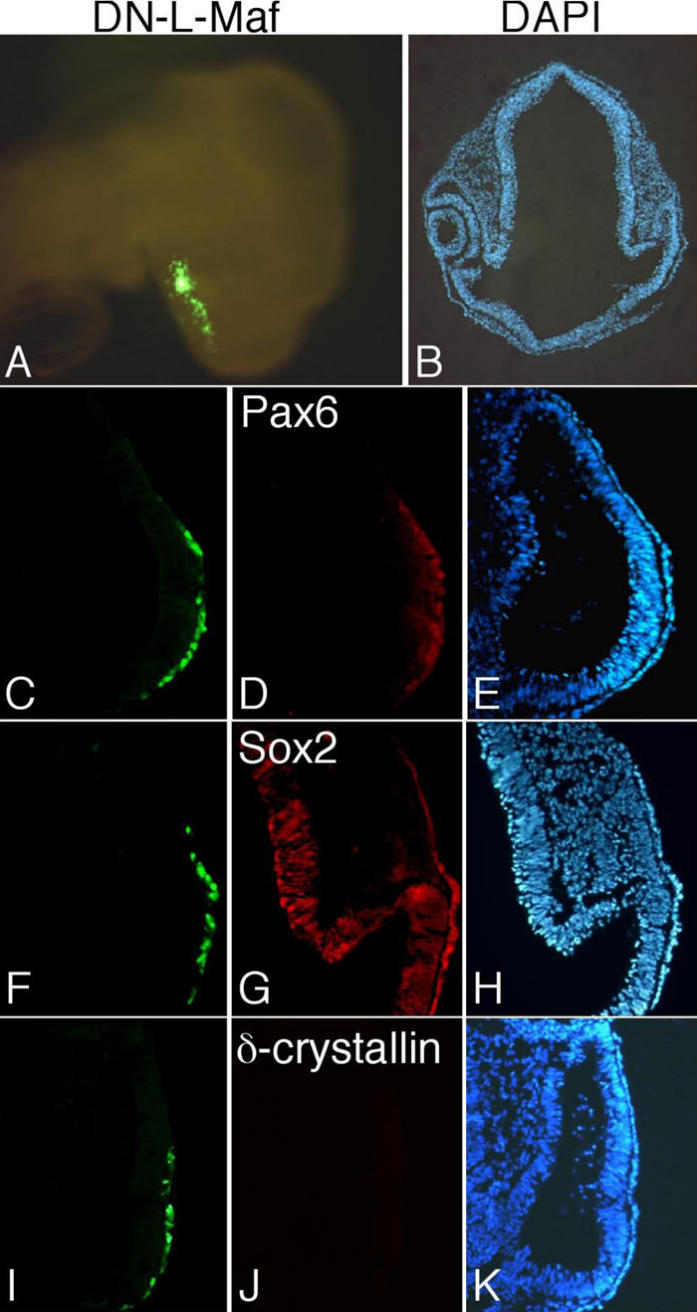
Lens formation is not initiated in the absence of L-Maf. A stage 16 whole embryo electroporated with dominant-negative L-Maf (DN-L-Maf) is shown (**A**). A section of this embryo showed no lens structure as visualized by DAPI staining (**B**). Immunohistochemical detection of Pax6 (**C**-**E**), Sox2 (**F**-**H**), and δ-crystallin (**I**-**K**) in the electroporated eye at stage 16. Expression of Pax6 (**D**) and Sox2 (**G**) was normal in the overlying surface ectoderm expressing dominant-negative L-Maf; however, δ-crystallin was not observed (**J**). GFP fluorescence indicates the electroporated area (**A**,**C**,**F**,**I**) and DAPI stains nuclei of the cells (**B**,**E**,**H**,**K**). This figure is representative of at least three independent experiments.

We demonstrated previously that L-Maf is essential for lens formation in chick, as overexpression of a dominant-negative L-Maf resulted in no lens structures; however, expression of two important genes, *Pax6* and *Sox2* was not investigated in that study [6]. To determine this, we performed an experiment using the same dominant-negative L-Maf to block endogenous L-Maf activity. By in ovo electroporation, we introduced dominant-negative L-Maf into the overlying head ectoderm of stage 9-10 chick embryos. Following a further 24 h of incubation, we isolated the embryo (Figure 3A) and examined the expression of Pax6, Sox2 and δ-crystallin (Figure 3C-K, stage 16). Immunostaining for Pax6 and Sox2 revealed their regular expression in the dominant-negative L-Maf-expressing cells of the ectoderm (Figure 3D,G). In contrast, we failed to detect the lens-specific marker δ-crystallin in the overlying ectoderm (Figure 3J). No lens structure developed in the electroporated eye, as determined by DAPI staining (Figure 3B). We did not observe invagination of the optic vesicle to form the optic cup, even though neural tissue was not manipulated. In the nonelectroporated contralateral eye, expression of all three markers was normal and the lens had normal morphology (Figure 3B and data not shown).

### Disparity in δ-crystallin induction by different Maf proteins

**Figure 4:**
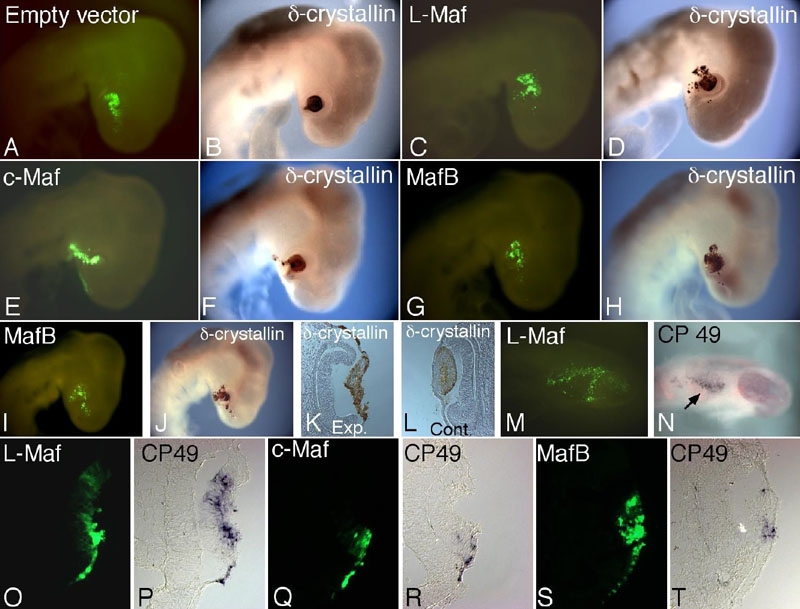
In vivo detection of δ-crystallin and CP49 by overexpressing L-Maf, MafB, and c-Maf, showing different inducing ability. Whole-mount immunostaining using δ-crystallin antibody was performed using stage 16 embryos electroporated with empty vector as negative control (**A**,**B**), wild-type L-Maf (**C**,**D**), c-Maf (**E**,**F**), and MafB (**G**-**L**) at stage 10. Embryo electroporated with empty vector showed no expression of δ-crystallin outside lens (**B**). Ectopic δ-crystallin expression was detected in all Maf-expressing embryos (**D**,**F**,**H**,**J**). However, it is apparent that L-Maf did induce δ-crystallin maximally (**D**). In most cases, MafB overexpression hampered lens shape (**J**,**K**). A section of the embryo shown in **J** revealed a lack of lens invagination and proper arrangement of cells (**K**). Nonelectroporated contralateral eye section showed normal expression of δ-crystallin and proper lens development (**L**). In similar experiments, cryosections from the eclectroporated embryos were subjected to in situ hybridization using Dig-labeled CP49 probe (**O**-**T**). L-Maf (**O**,**P**) strongly induced CP49, while c-Maf (**Q**,**R**) and MafB (**S**,**T**) showed lower activation of CP49. Further, whole-mount in situ hybridization was performed using L-Maf electroporated embryos after 5 h of incubation (**M**,**N**; stage 11), which revealed ectopic CP49 expression (**N**; arrow). Green fluorescence of GFP indicates transgene expression (**A**,**C**,**E**,**G**,**I**,**M**,**O**,**Q**,**S**). The figure is representative of at least three independent experiments. For whole-mounts, five to six embryos were used each time.

Accumulation of crystallins is an important event in lens development. To define the transactivation potential of L-Maf, c-Maf, and MafB in terms of downstream gene expression, we first investigated the expression of δ-crystallin in embryos electroporated with these genes by in ovo electroporation. δ-Crystallin is the first member of its kind to be expressed in chick lens. We observed ectopic expression of δ-crystallin when any *maf* gene was misexpressed in the surface ectoderm outside the lens-forming area of chick embryos (Figure 4C-J), which was also previously demonstrated for L-Maf only [6]. By comparing δ-crystallin-positive cells and GFP-positive cells, we found that the highest amount of δ-crystallin expression was yielded with L-Maf (Figure 4C,D) and the lowest was detected with c-Maf misexpression (Figure 4E,F). Histology clearly revealed that overexpression of L-Maf and c-Maf in presumptive lens ectoderm did not perturb the normal cellular arrangement in the lens placode (data not shown). In contrast, lens cells were not properly organized to form the regular lens vesicle; rather, they seemed to adhere to the overlying ectoderm when MafB was overexpressed in the presumptive lens cells (Figure 4K). A section of the non-electroporated contralateral eye exhibited normal development of lens (Figure 4L).

### Lens-specific genes are upregulated by Maf proteins to varying levels

To characterize the regulatory ability of Maf on lens-expressing genes in more detail, we investigated the expression profile of the lens-fiber specific genes *cp49* and *cp95* upon misexpression of L-Maf, c-Maf, and MafB in the ectoderm of stage 10 chick embryos. From our whole-mount (data not shown) and cryosection in situ hybridization, we found that all three members were able to induce both CP49 (Figure 4M-T) and CP95 (data not shown). L-Maf could strongly activate CP49 (Figure 4O,P) and 95 in most of the cells that were electroporated. In addition, in situ hybridization revealed ectopic expression of CP49 transcripts in the L-Maf-expressing cells only 5 h after electroporation (Figure 4M,N). On the other hand, c-Maf and MafB could induce these genes only to a lower extent (Figure 4Q,R and Figure 4S,T, respectively).

### Differential effect of Maf on cadherin and Six3 expression in lens lineage

**Figure 5:**
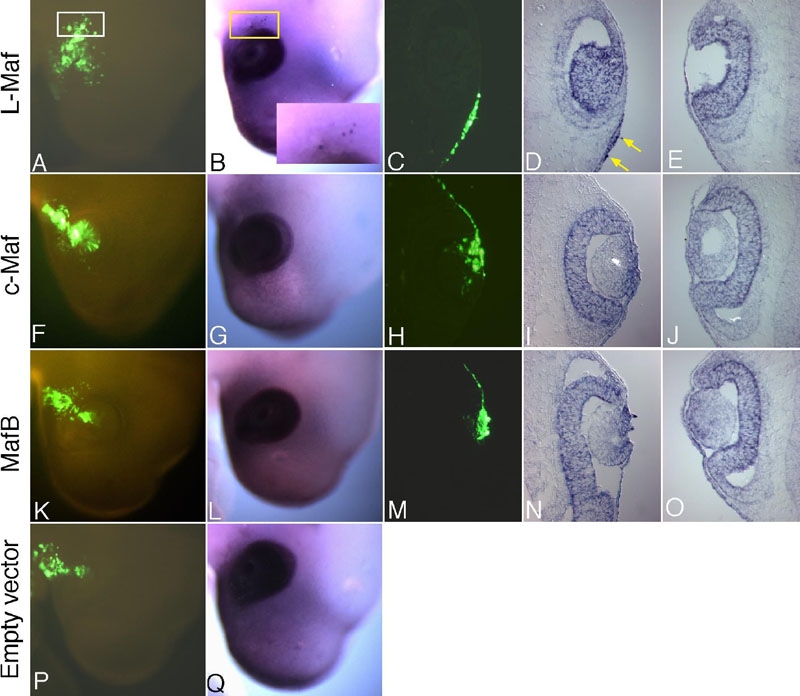
Whole-mount and cryosection in situ hybridization exhibit ectopic expression of Six3 when L-Maf, but not c-Maf and MafB, is misexpressed. Electroporated chick embryos with L-Maf (**A**-**E**), c-Maf (**F**-**J**), MafB (**K**-**O**), and empty vector (**P**,**Q**) were analyzed whole and in sections at stage 16 to follow the expression of Six3. Ectopic Six3 was detectable in the surface ectoderm expressing exogenous L-Maf (**B**; yellow box shown in the inset; **D**; yellow arrows), but undetectable in c-Maf (**G**,**I**) and MafB (**L**,**N**) expressing cells outside lens. Contralateral nonelectroporated eyes showed normal expression of Six3 (**E**,**J**,**O**). Embryo electroporated with empty vector as control showed only endogenous expression of Six3 (**Q**). Green fluorescence of GFP depicts electroporated cells (1st and 3rd columns). This figure is representative of at least three independent experiments. For whole-mounts, five to six embryos were used each time.

A previous study showed that Six3 overexpression causes the inhibition of lens placode invagination and persistence of the placodal state in isolated groups [32], which can be compared phenotypically with the effect obtained by MafB overexpression in this study (Figure 4J,K). To test whether or not MafB function is linked to Six3, we examined expression of Six3 in MafB-electroporated embryos (Figure 5K-O). MafB failed to produce any effect on Six3 expression, as detected by in situ hybridization using a probe for Six3 (Figure 5L,N). As a negative control, we examined the expression of Six3 using an embryo that was electroporated with empty vector and found no ectopic expression (Figure 5P,Q). While we were examining the effect of MafB on Six3, we looked at its expression in L-Maf- and c-Maf-electroporated samples (Figure 5A-J). We found c-Maf showed an identical effect to that of MafB on Six3 expression (Figure 5G,I). In contrast, L-Maf induced ectopic expression of Six3 mRNA in the overlying surface ectoderm as determined by whole-mount and cryosection in situ hybridization (Figure 5B,D, boxes and arrows). However, no notable change in cell shape or arrangement was observed. Contralateral nonelectroporated eyes showed regular expression of Six3 (Figure 5E,J,O).

**Figure 6:**
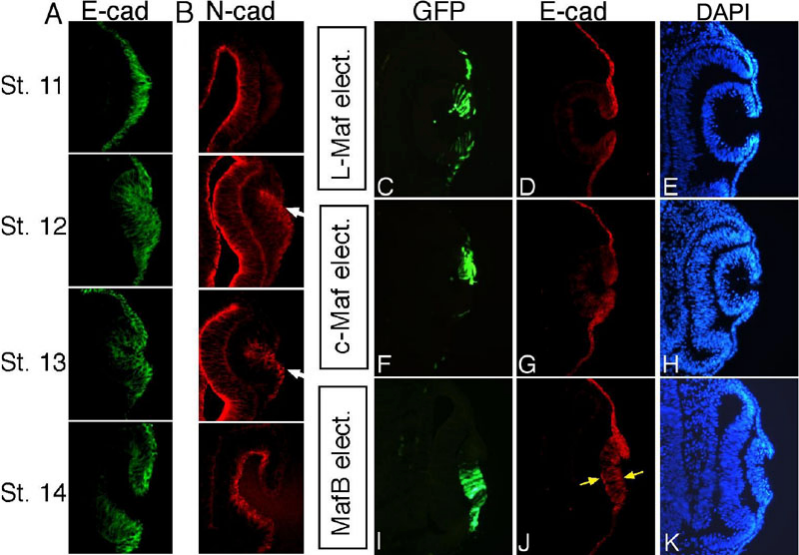
Effects of Maf expression on E-cadherin. Expression patterns for E- and N-cadherins by section immunostaining are shown at stage 11, 12, 13, and 14 (**A**,**B**, respectively). White arrows indicate the N-cadherin-positive cells (**B**). Immunohistochemical analyses with the cryosections from stage 16 embryos revealed ectopic expression of E-cadherin in the invaginating lens placode when MafB was electroporated (**J**, yellow arrows). No effect on E-cadherin is observed in the sections electroporated with L-Maf (**D**) and c-Maf (**G**). DAPI visualized cell nuclei (**E**,**H**,**K**). Green fluorescence of GFP depicts electroporated cells (**C**,**F**,**I**). This figure is representative of at least three independent experiments.

Next, we examined the expression of two adhesion molecules, E- and N-cadherin, in Maf-electroporated eyes. It has been demonstrated that cell movement and fate determination occur with the altered expression of different *cadherin* genes during vertebrate development [33-35]. First, we studied the endogenous expression of these two genes in a narrow time frame ranging, from stage 11 to stage 14 (Figure 6A,B). Immunostaining analyses showed that E-cadherin is extensively expressed and restricted in the ectodermal epithelial cells, and that the expression declines in the invaginated lens cells (Figure 6A). N-cadherin expression is initiated at the onset of placode cell invagination (Figure 6B, arrows) and is absent from the ectodermal epithelium (Figure 6B). Overexpression of MafB resulted in enhanced accumulation of E-cadherin protein in the partially invaginated lens placode forming the lens vesicle (Figure 6J, arrows), but N-cadherin was unaltered (data not shown). Immunostaining using anti E- and N-cadherin revealed no change in expression when L-Maf (Figure 6C-E) or c-Maf (Figure 6F-H) or empty vector (data not shown) was overexpressed.

### Connexins and MIP are regulated by Maf

**Figure 7:**
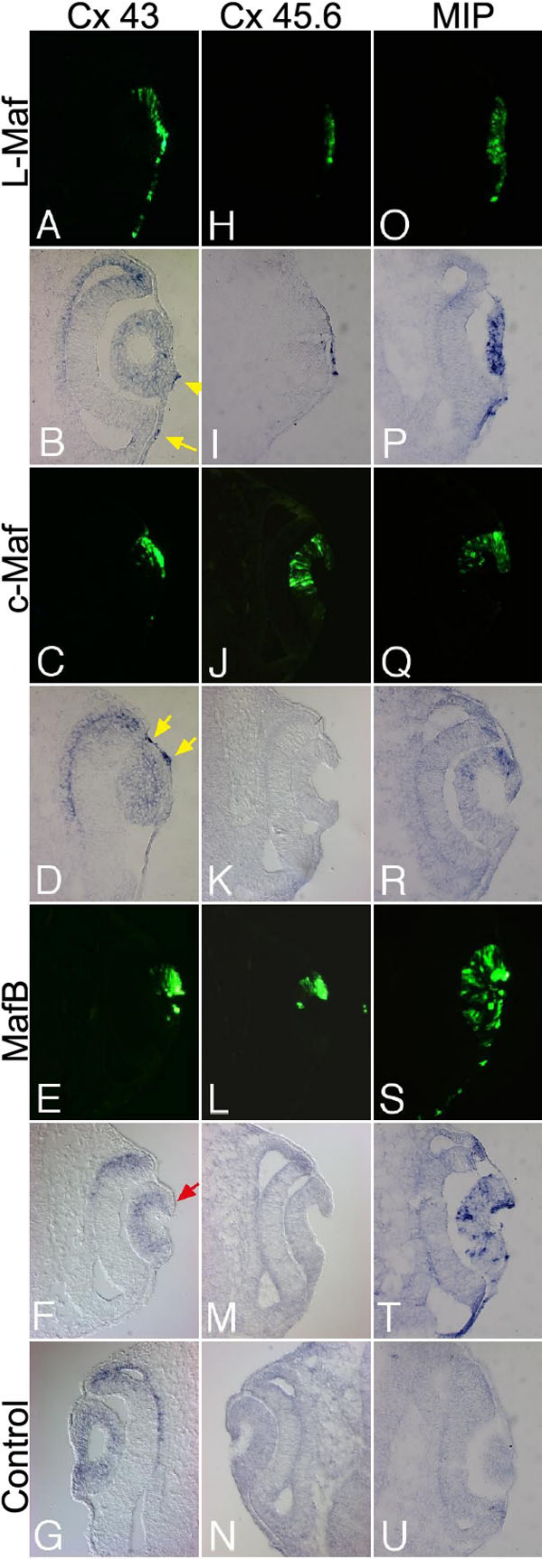
Maf misexpression stimulates Cx43, Cx45.6, and MIP in electroporated cells. Cryosections prepared from stage 16 embryos electroporated with wild-type L-Maf, c-Maf, and MafB were subjected to in situ hybridization with Cx43 (**A**-**G**), Cx45.6 (**H**-**N**), and MIP (**O**-**U**). Both L-Maf and c-Maf induced the expression of Cx43 (**B**,**D**, respectively, yellow arrows), while MafB suppressed Cx43 expression in the electroporated cells (**F**, red arrow). Cx 45.6 was activated by L-Maf only (**I**). c-Maf and MafB did not induce Cx45.6 (**K**,**M**, respectively). L-Maf induced the highest expression of MIP (**P**), followed by MafB (**T**) and c-Maf (**R**). Nonelectroporated contralateral eyes revealed that Cx45.6 (**N**) and MIP (**U**) were not expressed at this stage, while regular expression of Cx43 (**G**) was detected. Green fluorescence (GFP) indicates transgene expression (**A**,**H**,**O**,**C**,**J**,**Q**,**E**, **L**,**S**). This figure is representative of at least three independent experiments.

Since gap junction proteins such as connexins are abundantly expressed in lens during development [36-38], we were interested to elucidate if there were a link between Maf and connexin as well as their interacting partners. Among three connexins identified in chick lens, Cx43 is preferentially expressed in epithelial cells, whereas Cx45.6 is expressed in fiber cells [23,37]. Therefore, we analyzed the expression of these two members by in situ hybridization, following electroporation with L-Maf, c-Maf, and MafB, to address the effect of Maf on these genes (Figure 7). Section in situ hybridization showed that L-Maf (Figure 7A,B, yellow arrows) and c-Maf (Figure 7C,D, yellow arrows) can induce ectopic Cx43 expression when misexpressed in the overlying surface ectoderm. On the other hand, MafB-expressing populations showed an inhibition of Cx43 expression (Figure 7E,F, red arrow). The other connexin, Cx45.6, is activated mainly by L-Maf (Figure 7H,I), while c-Maf and MafB have little or no effect (Figure 7J-N). We also monitored the expression of a later-expressed gene, *MIP*, in differentiating lens fiber. It has been reported that overexpression of Cx45.6 increases MIP expression in primary lens cultures and stimulates differentiation [25]. We observed that L-Maf and MafB induced MIP (Figure 7O,P and Figure 7S,T, respectively) in the invaginating lens placode, while c-Maf had little effect on MIP expression (Figure 7Q,R). In the nonelectroporated contralateral lens, Cx45.6 and MIP were not detected but Cx43 was found (Figure 7N,U,G, respectively), which was expected as MIP and Cx45.6 are not expressed at this stage.

## Discussion

In this study, we performed overexpression experiments in which three *maf* genes were electroporated into the overlying surface ectoderm of chick embryos at stage 10 by in ovo electroporation. The electroporated embryos were then isolated at stage 16, and subsequent analysis was performed to understand the effects of the *maf* genes on the expression of various downstream genes in lens by in situ hybridization and immunostaining. Finally, these results have been correlated with the expression patterns of the genes examined, and discussed in relation to the normal course of lens development. A regulatory relationship among the large Mafs is established by their order of expression

Expression pattern analysis of the *maf* genes has confirmed that three members of the large Maf family are expressed during lens development in the chick [7,39]. Our close observation has revealed that L-Maf is expressed earliest in the presumptive lens ectoderm immediately after contact between the optic vesicle and overlying surface ectoderm [7]. c-Maf expression starts only a few hours later than L-Maf expression, while MafB is expressed much later in the developing lens. The overexpression experiments detailed here (Figure 2) show that a simple regulation mechanism is present among three large *maf* genes, in which an early-expressed Maf positively regulates the transcription of later-expressed one(s). Combining this data with the expression patterns of these three *maf* genes, we propose that this regulatory hierarchy is established by their endogenous order of expression and that it possibly functions during the normal course of lens development, thus signifying distinct biological functions for different large Maf proteins in successive stages of lens development as transactivators of temporal and region-specific genes. We did not find gene activation in the reverse direction by overexpression experiments but we did find that later-expressed MafB negatively regulates the early-expressed L-Maf or c-Maf (Figure 2U-Z2 [8], ). It is possible that gene regulation within the *maf* family may occur through the MARE sequence. A previous study sheds light on this, demonstrating that the *c-maf* gene is autoregulated by its own product through MARE [40].

We have found that expression of exogenous MafB in presumptive lens cells before the onset of the endogenous gene's expression perturbs the genesis of the lens. However, it could activate δ-crystallin (Figure 4I-K), suggesting that timely expression of Maf proteins is critical for lens development, presumably transcription of different sets of genes that govern accurate lens formation.

### L-Maf propagates the lens-inductive roles of Pax6 and Sox2

Previous studies have demonstrated that Pax6 and Sox2 are two key factors that initiate lens development in mouse and chick embryos [5,6]. Several lines of evidence show that L-Maf is a unique transcription factor that plays a major role in lens induction in the chick. The inhibition of endogenous gene function achieved by expressing a dominant-negative form, described here, is a useful tool with which we have uncovered an important issue regarding this process. In our overexpression experiments, Pax6 and Sox2 proteins were detectable in the surface ectoderm when L-Maf function was inhibited by overexpressing a dominant-negative form of L-Maf. On the other hand, δ-crystallin was absent, as indicated by immunostaining (Figure 3J). These data suggest that the lens-specific marker δ-crystallin is not expressed, despite the presence of two essential regulators, unless L-Maf participates. We propose that the early driving force for lens initiation from surface ectoderm generated by Pax6 and Sox2 is transmitted through L-Maf, thus L-Maf functions downstream of Pax6 and Sox2 to activate δ-crystallin in processes leading to lens induction. Our previous finding that a synergistic effect of Pax6 and Sox2 ectopically activates L-Maf strongly supports this notion. The presence of putative binding sites for Pax6 and Sox2 in the 5'-upstream sequence of the *L-maf* gene, and an L-Maf binding site in the core sequence of the δ-crystallin enhancer, similarly argue for this hypothesis (for a review, see [11]). However, since we cannot exclude the possibility that our dominant-negative L-Maf may inhibit the function of c-Maf or other Maf-interacting factors, we suggest that Pax6 and Sox2 synergistically induce δ-crystallin expression by activating Maf during lens induction in chick embryos. In relation to comparative Maf activity in chick and mouse, c-Maf does not take part in mouse lens induction but functions in fiber cell differentiation [12-14]. L-Maf/MafA has not yet been identified in mouse lens. Such apparent functional asymmetry may be explained simply by species variation.

### Potential to transactivate downstream genes varies among Maf proteins

Large Maf proteins share the common property of inducing crystallin. This has been supported by our in ovo electroporation studies using *maf* genes from different species (H.M.R. and K. Kataoka, unpublished data). Misexpression of human, mouse, and chick MafA/L-Maf induces δ-crystallin in chick embryos, and although MafA, the mammalian homolog of chick L-Maf, has not yet been detected in mouse or human developing lens, two other members of this family, c-Maf and MafB, are expressed later in development.

We have found that L-Maf, c-Maf, and MafB are capable of eliciting δ-crystallin expression when overexpressed. However, they do so to varying degrees, as indicated by whole-mount immunostaining data (Figure 4C-H). These results, together with expression studies on Maf, suggest that L-Maf probably restricts its ultimate activity to the fiber cells, while c-Maf is mainly responsible for regulating genes expressed in epithelial cells. MafB is likely to regulate later-expressed crystallins and other unidentified genes in the developing lens. Our current in vivo data essentially concur with previous observations using cultured cells, confirming that crystallin-inducing ability differs for each of the Maf members [8]. Hence, crystallin regulation is likely to be shared by different Maf members and is presumably determined by developmental stage and the availability of upstream regulatory factors.

Similarly, L-Maf can ectopically induce the noncrystallin genes *cp49* and *cp95/115* when misexpressed in cultured cells or chick embryos [4] (Figure 4M-P). The lens of *c-maf* homozygous knockout mice showed an absence or impaired expression of CP49 and CP115 as detected by immunohistochemical experiments [21]. These findings demonstrate that Maf proteins regulate the expression of these beaded filament genes. The levels of mRNA observed by in situ hybridization using Maf-electroporated sections in this study indicate that these genes are not activated equally by all Maf proteins. It is probably dependent on their relative roles and, to some extent, on the availability of cofactors. The highest activity was found with L-Maf (Figure 4O,P). While a previous study suggested an indirect regulation of *cp49* and *cp95* genes by c-Maf in mice, as shown by a reporter assay using NIH3T3 cells [21], our observation that L-Maf induces CP49 5 h after electroporation in the surface ectoderm of chick embryos (Figure 4M,N) indicates that CP49 induction may be a direct consequence of L-Maf misexpression in chick. Further in vitro studies will be required to confirm this. Since c-Maf expression is more favored in epithelial cells in chick, and gain-of-function experiments with c-Maf have revealed minimal expression of CP49 and CP115, we think that c-Maf has little effect on these genes during normal lens development. However, as we performed overexpression experiments using the same-stage embryos, we found it possible that the lack of some cofactor important for c-Maf or MafB function at particular stages may also influence the results.

N-cadherin expression begins in placode cells at the time of invagination (Figure 6B, arrows), indicating that N-cadherin may function in cell movement during placode invagination. We found that E-cadherin, which is characteristically expressed in ectodermal cells, was upregulated by MafB electroporation as shown by immunostaining (Figure 6I-K), and MafB overexpression hampered proper lens vesicle formation (Figure 4K). We assume that increased E-cadherin expression by MafB may hinder the progression of lens placode invagination and cell movement to form a proper lens vesicle, and we therefore suggest that a low level of MafB expression in lens epithelium may confer stability to E-cadherin and maintains the integrity of epithelial cells during the normal course of development. The epithelium-preferred expression of Six3 was unaltered by both MafB and c-Maf overexpression, but L-Maf increased Six3 expression substantially (Figure 5A-D). These results demonstrate that L-Maf lies upstream of Six3 in lens epithelium, whereas the other two Mafs probably do not interact with Six3 expression. All these observations suggest that Maf proteins also drive the expression of genes in lens epithelium, each in a distinct manner.

As development proceeds, c-Maf is preferentially confined to the lens epithelium while L-Maf resides in the differentiating lens fibers with a high level of expression in the bow region. MafB expression is detected at stage 26 in both epithelial and fiber cells [7]. This differential expression of *maf* family genes is consistent with our experimental findings, suggesting that different Maf proteins expressed in the same tissue have distinct functions at particular stages, and therefore that the spatiotemporal expression of Maf proteins within the developing lens defines their exact role in activating later downstream genes essential for lens development. A comprehensive quantitative analysis of the three large Mafs should reveal further useful information about the precise role of each Maf protein.

### Regulation of connexins and MIP as a novel function of Maf

Gap junction channels formed by different connexins are important for maintaining normal lens physiology. Maf proteins have not previously been shown to control any member of the connexin family. Since overexpression of Cx45.6 stimulated lens cell differentiation and augmentation of crystallins [25], we anticipated that *maf* gene products might interact with this class of genes during development. As observed from our experiments, induction of Cx43 and Cx45.6 by L-Maf overexpression (Figure 7A,B,H,I) implies that they are likely to be controlled by L-Maf both in lens fiber and in epithelial cells during normal lens development. Since both c-Maf and Cx43 are expressed more highly in epithelium than L-Maf, a higher level of Cx43 induction by c-Maf was found as expected (Figure 7A-D). On the other hand, Cx45.6 is mainly induced by L-Maf (Figure 7H,I), suggesting that the fiber-specific gene *Cx45.6* is mostly regulated by L-Maf, as L-Maf expression persists in fiber cells throughout lens development. MafB is weakly involved in the regulation of gap junction molecules in the lens. We propose that L-Maf, among the Maf proteins, plays the vital role to establish functional intercellular communication through connexins in lens fiber cells, while c-Maf may exert a similar function in epithelial cells.

MIP is preferentially expressed in differentiating fiber cells, and there is evidence that it interacts directly with CX45.6 in lens fiber cells [24]. Our gain-of-function experiment revealed that MIP is profoundly activated by L-Maf and MafB (Figure 7O,P,S,T), which is logical as these two proteins are abundantly expressed in differentiating fiber cells. However, overexpression of c-Maf is also capable of inducing Cx45.6; this also appears to be possible as c-Maf is still observed in fiber cells to some extent. Our results demonstrate that L-Maf and MafB are more important in regulating fiber-specific membrane proteins than c-Maf. We speculate that L-Maf and c-Maf participate in intercellular communication by regulating connexins; on the other hand, MafB plays an important role in regulating MIP, whose function is still to be determined. It may be assumed that MafB-regulated MIP might have some additive characteristics within the higher mass of elongated fiber cells in the lens. Indeed, Maf proteins function at different stages of development, so we cannot rule out the possibility that the observed downstream effects of *maf* genes have not fully addressed their endogenous functions since these conclusions are based on overexpression experiments using same-stage embryos.

**Figure 8:**
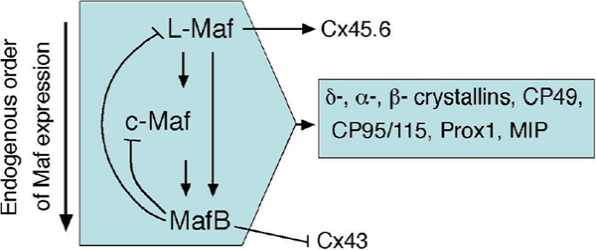
A diagram showing gene regulation within the *maf* family and of other effector genes during lens development in the chick. The bold arrow indicates the order of expression for the three large *maf* genes in lens lineage. An early-expressed Maf positively regulates later-expressed members. Later-expressed MafB inhibits the expression of L-Maf and c-Maf. All three Maf proteins can activate several common genes such as *δ-crystallin*, *cp49*, *cp95*, *Prox1* [6], (H.M.R. and K.Y., unpublished data), and *MIP*. On the other hand, distinct effects are also observed, as L-Maf induces Six3, Cx43, and Cx45.6; c-Maf upregulates Cx43, and MafB induces E-cadherin but downregulates Cx43. Hence, both redundant and distinct functions of L-Maf, c-Maf, and MafB essentially control many vital genes during lens development.

In summary, we conclude that large Maf family transcription factors constitute a specific regulatory network during lens development (Figure 8). The integrated functions, some redundant and some parallel, exerted by different Mafs drive lens development and ensure the normal physiology of a developing lens by regulating genes of diverse families such as those encoding transcription factors, crystallins, non-crystallins and membrane proteins. It will be of great interest to determine the molecular mechanisms of how different Maf proteins regulate this wide range of target genes.
